# DESIGN AND MODELING FOR SPARSELY SAMPLED PHYSIOLOGICAL SYSTEMS DATA

**DOI:** 10.1093/geroni/igae098.3290

**Published:** 2024-12-31

**Authors:** Charlotte Clapham, Ravi Varadhan, Karen Bandeen-Roche

**Affiliations:** Johns Hopkins University Bloomberg School of Public Health, Baltimore, Maryland, United States; Johns Hopkins University, Baltimore, Maryland, United States; Johns Hopkins University, Baltimore, Maryland, United States

## Abstract

Physical resilience is a pressing research area in aging. The Study of Physical Resilience and Aging (SPRING) posits that resilience is rooted in the fitness of specific physiological systems: It is studying resilience by introducing stressors on selected systems and monitoring their return to homeostasis as time series. We aim to use data from these dynamical stimulation tests to model the fitness of underlying dynamical systems; However, the extreme sparsity and measurement error in SPRING presents a methodological challenge. This presentation reports studies to evaluate alternative methods for recovering system dynamics. We first explored the limits of Local Linear Approximation (LLA) for this purpose using data simulated from curves sampled with varying levels of sparsity and measurement error. We found that LLA could not accurately or precisely recover the curves’ dynamics when data was extremely sparsely sampled or had moderate measurement error, making it unviable for SPRING. Our presentation also reports work to develop more nuanced methodologies that implement integro-differential equation modeling, smoothing techniques, and leveraging of ancillary data from other studies that performed dynamic stimulation tests on older adults. These strategies aim to enhance signal, reduce measurement error and borrow strength incorporating existing knowledge. This work has implications on our ability to better understand and predict resilience.

